# Supplementary figures and images for: miRNA activity inferred from single cell mRNA expression
Source: Sci Rep. 2021 Apr 28;11:9170. doi: 10.1038/s41598-021-88480-5 (PMC8080788; doi:10.1038/s41598-021-88480-5)

miR-122 expression

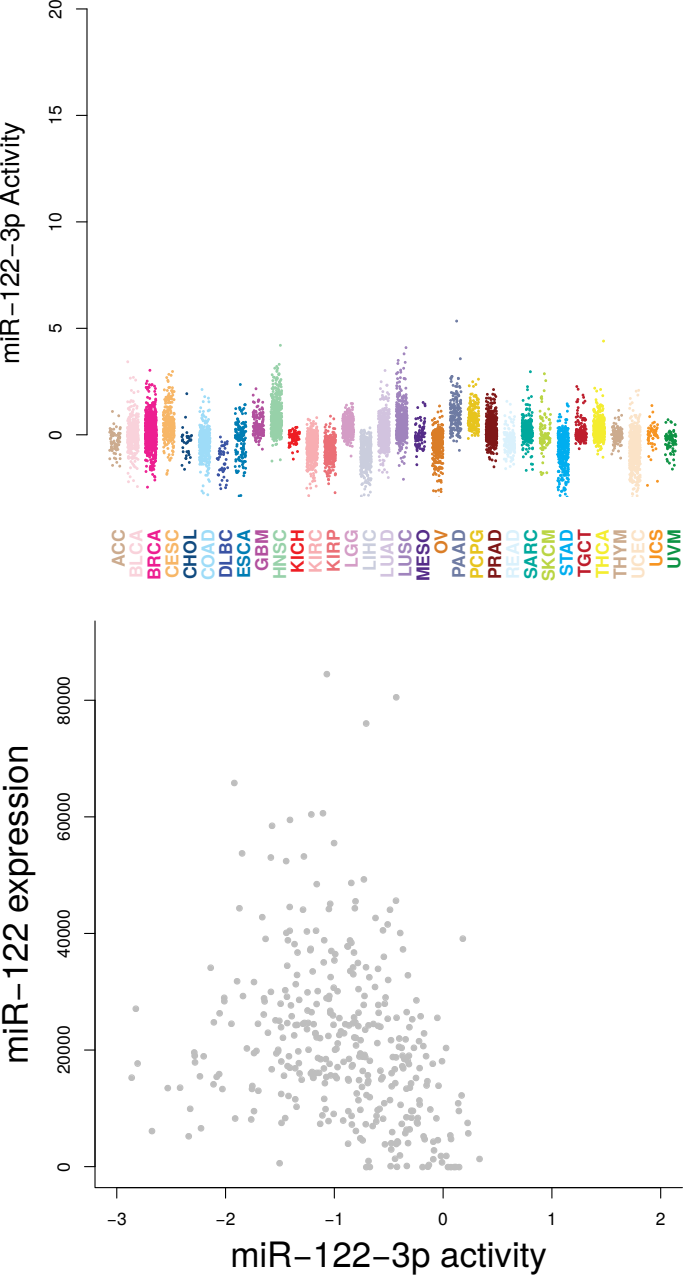

Supplement: Supplementary file 1 — Supplemental Figure 1. [file 41598_2021_88480_MOESM1_ESM.pdf]

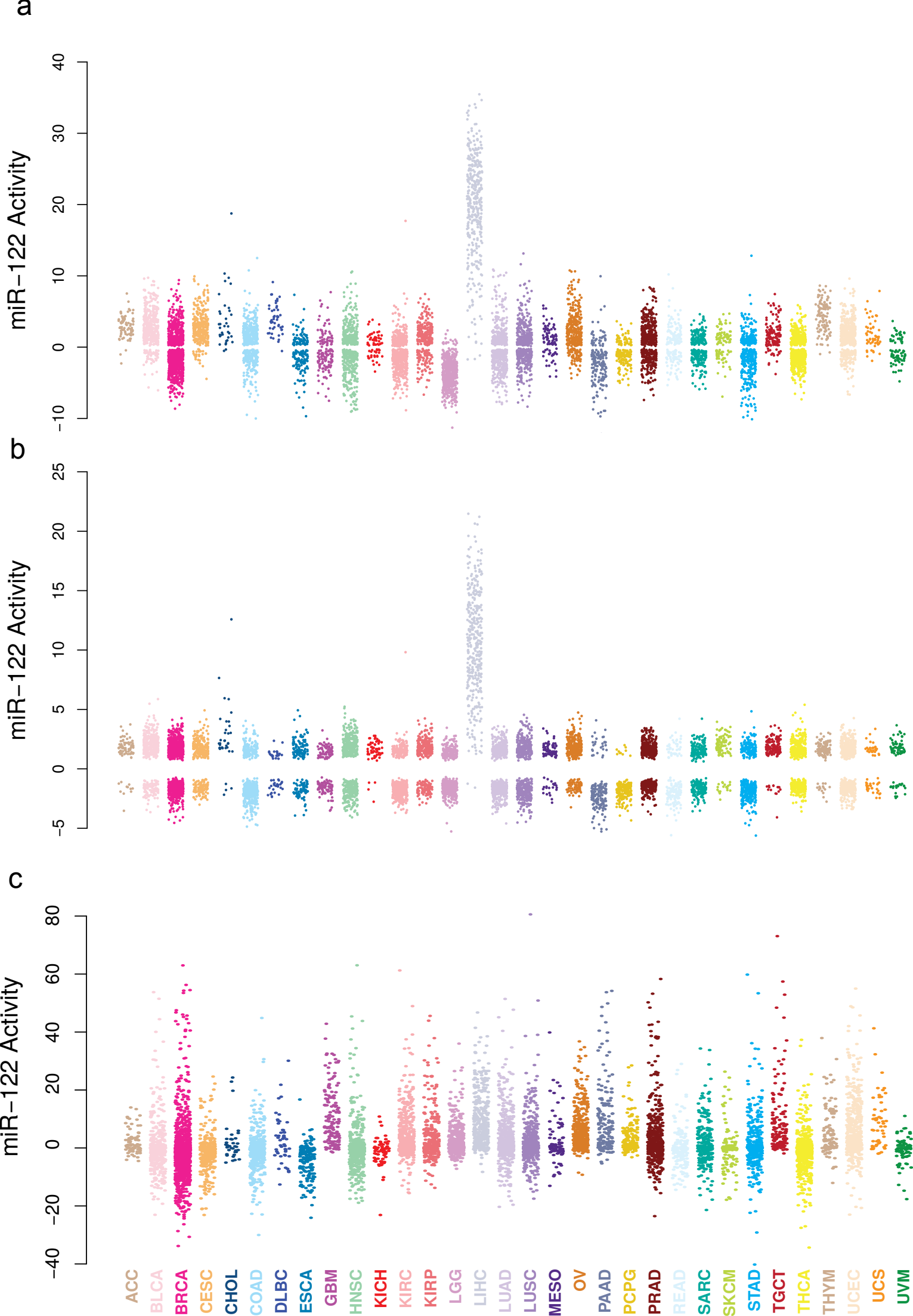

Supplement: Supplementary file 2 — Supplemental Figure 2. [file 41598_2021_88480_MOESM2_ESM.pdf]

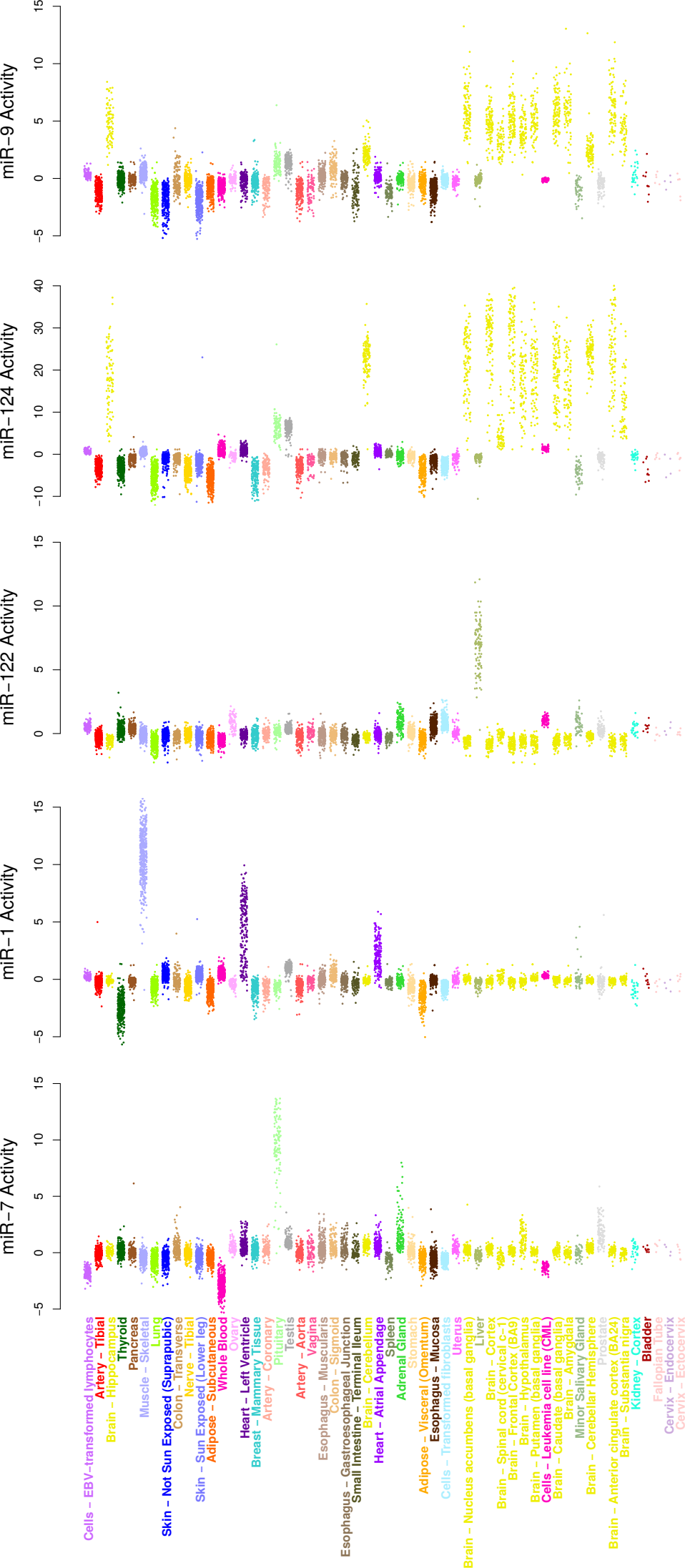

Supplement: Supplementary file 3 — Supplemental Figure 3. [file 41598_2021_88480_MOESM3_ESM.pdf]

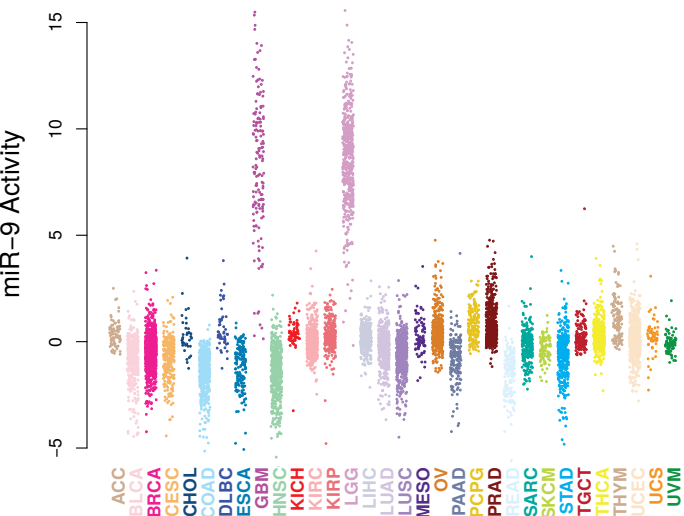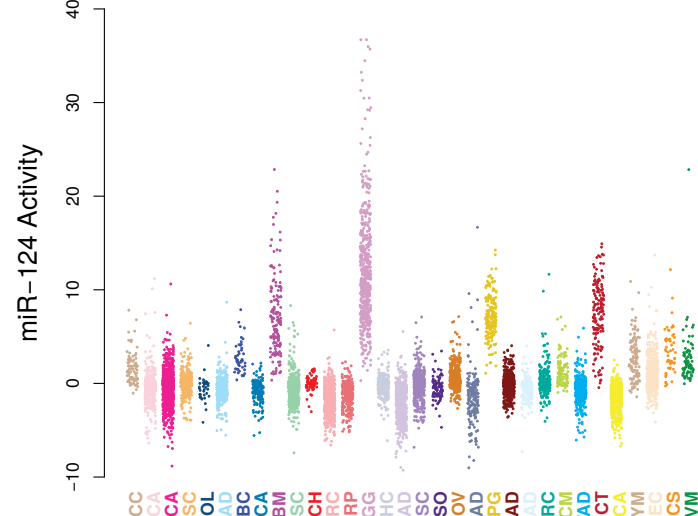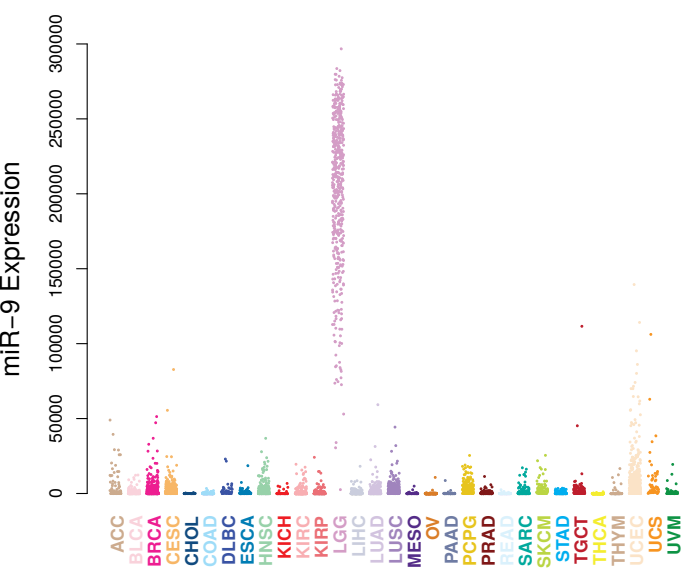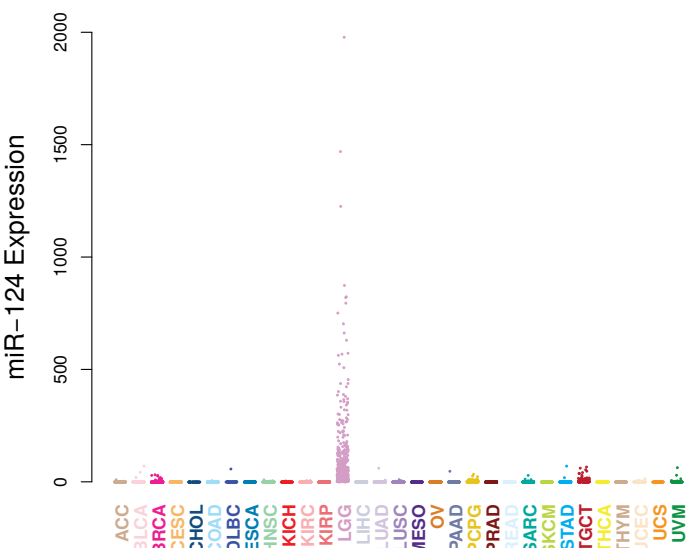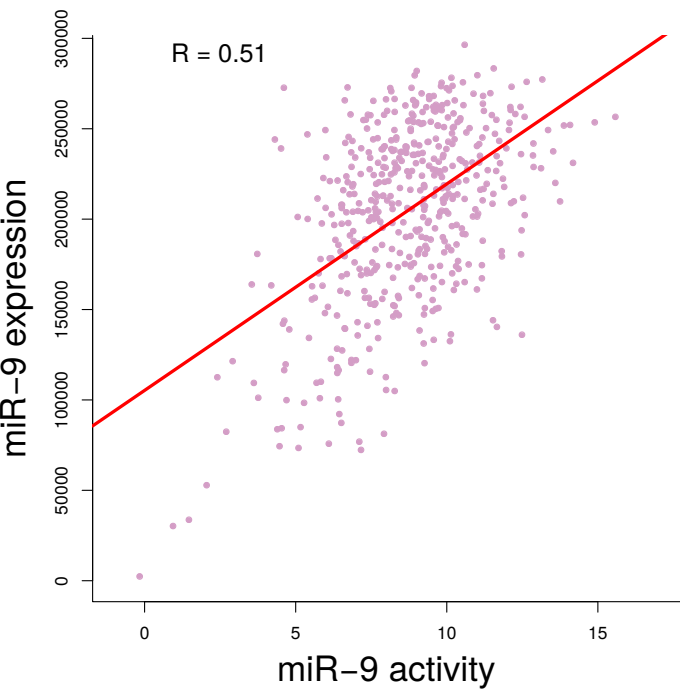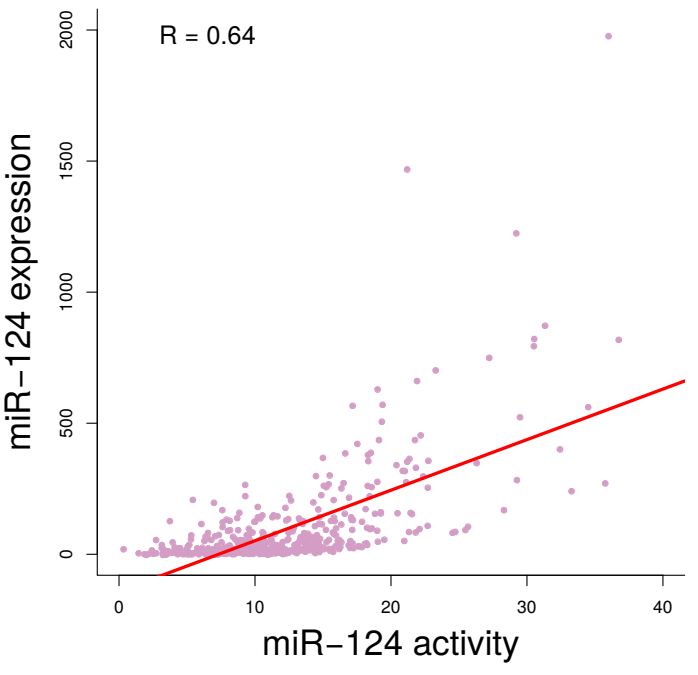

Supplement: Supplementary file 4 — Supplemental Figure 4. [file 41598_2021_88480_MOESM4_ESM.pdf]

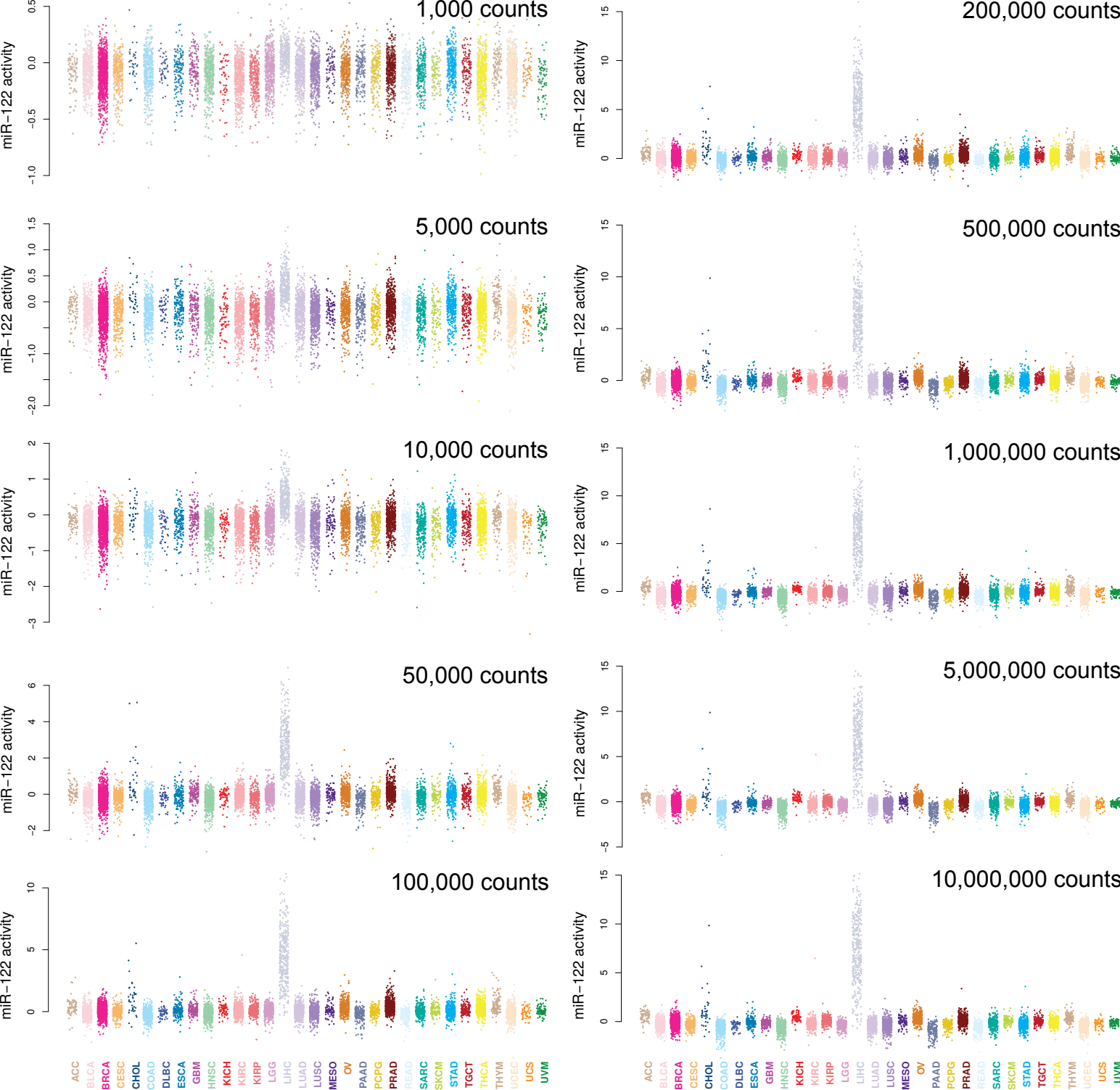

Supplement: Supplementary file 5 — Supplemental Figure 5. [file 41598_2021_88480_MOESM5_ESM.pdf]

# Tarbase gene rank sum vs mir122 activity

## Liver cells

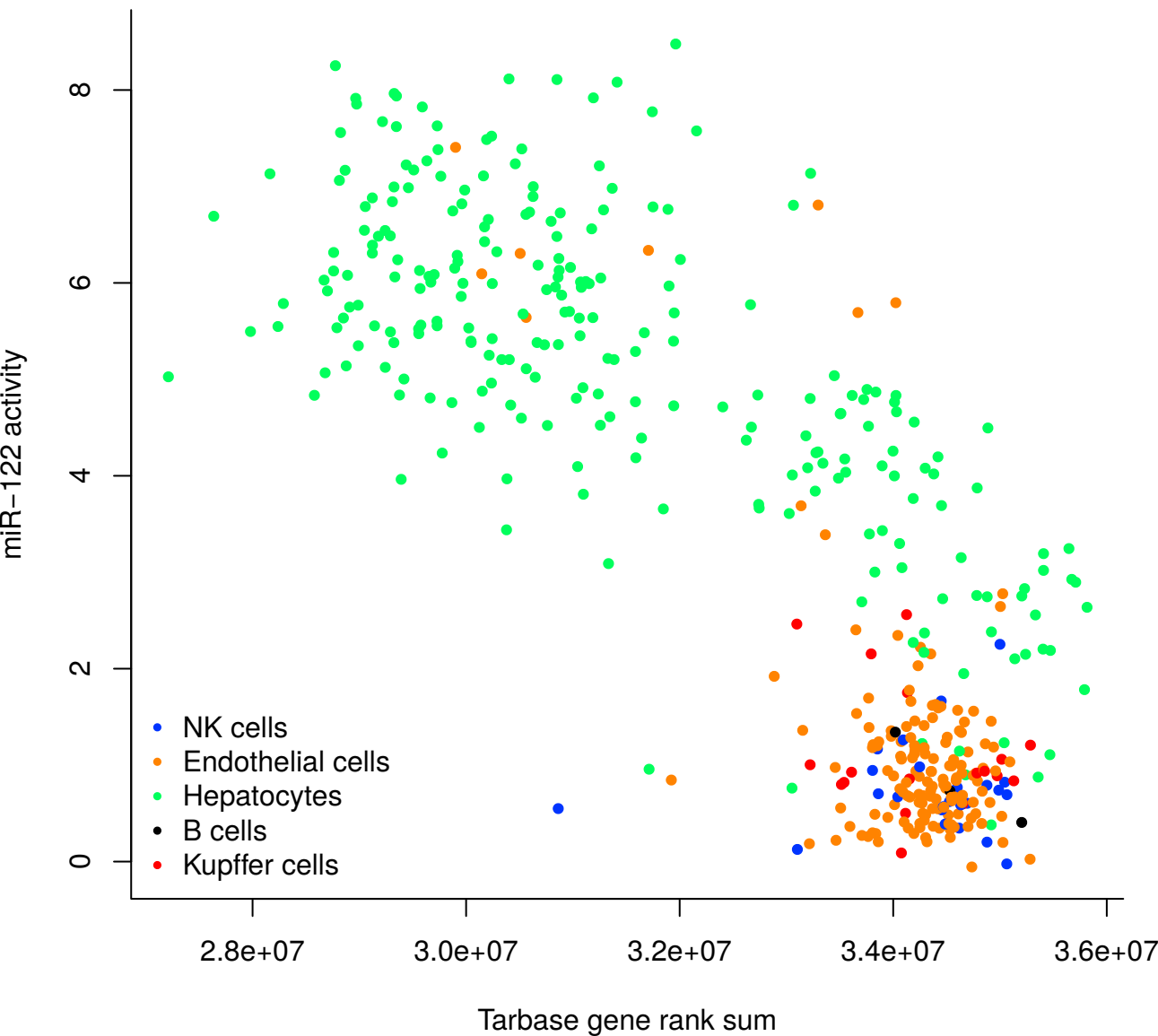

Supplement: Supplementary file 6 — Supplemental Figure 6. [file 41598_2021_88480_MOESM6_ESM.pdf]

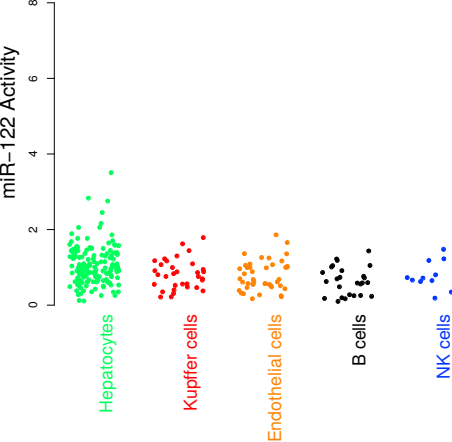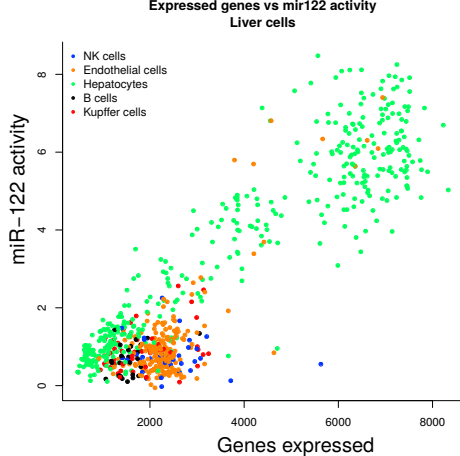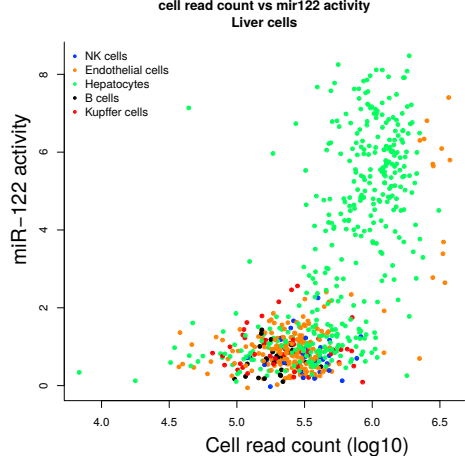

Supplement: Supplementary file 7 — Supplemental Figure 7. [file 41598_2021_88480_MOESM7_ESM.pdf]

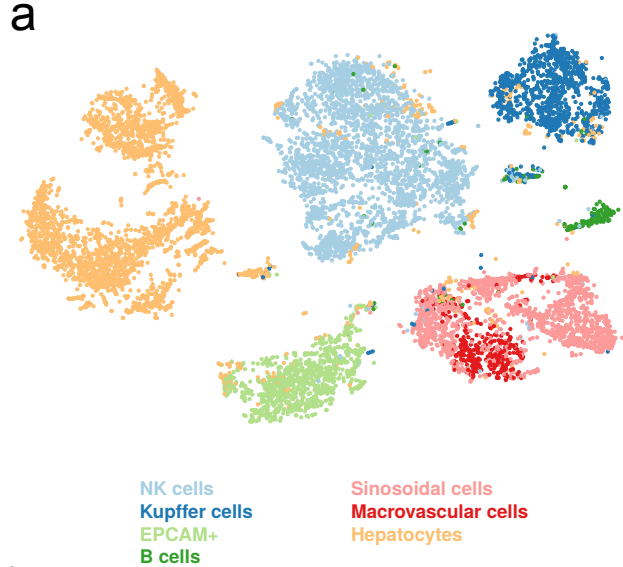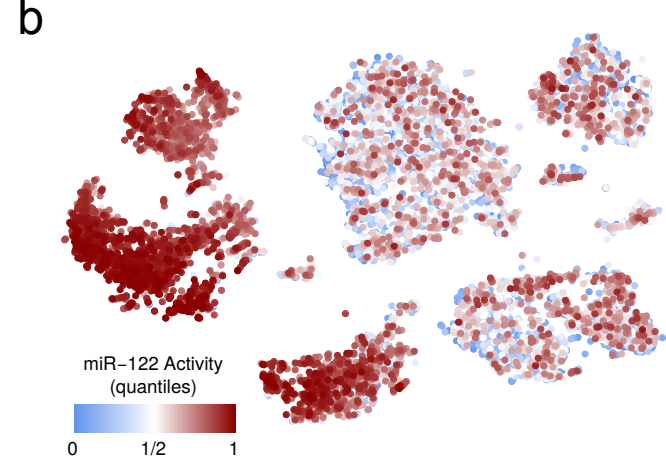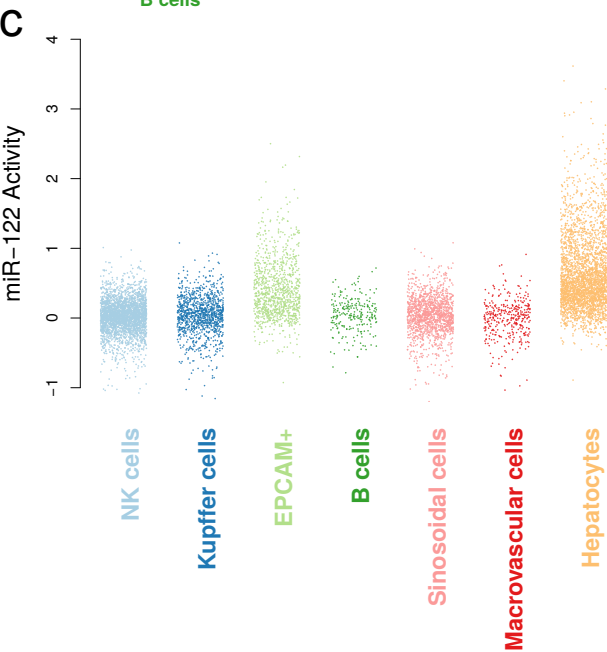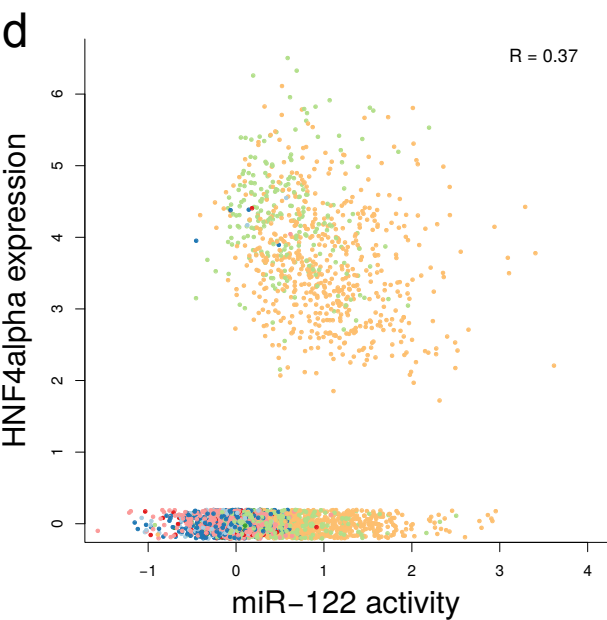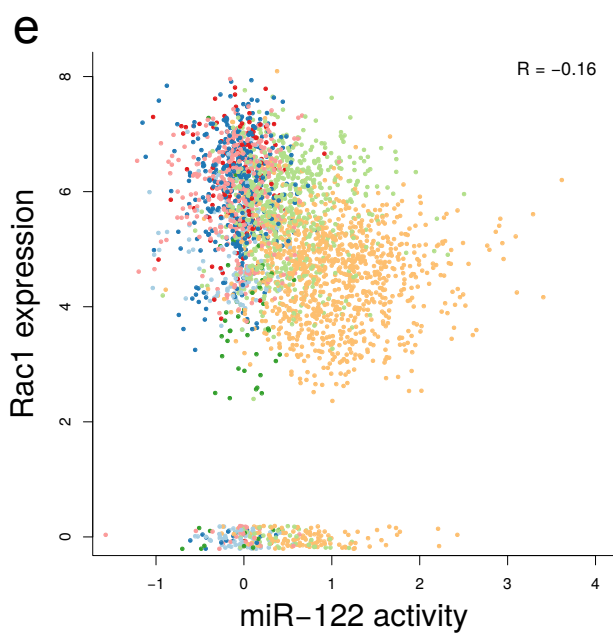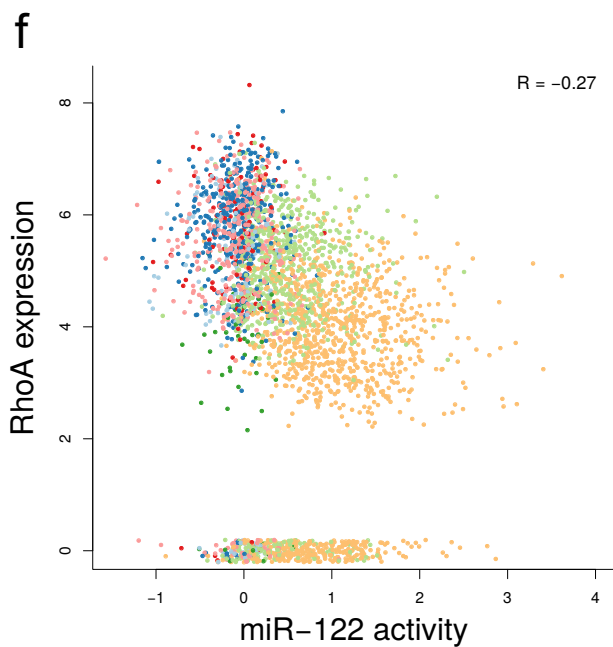

Supplement: Supplementary file 8 — Supplemental Figure 8. [file 41598_2021_88480_MOESM8_ESM.pdf]

**a**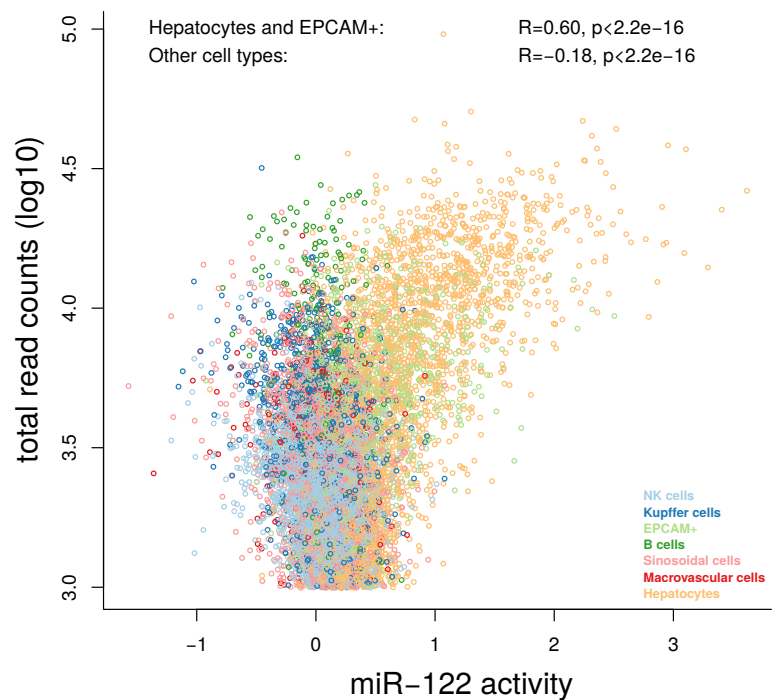**b**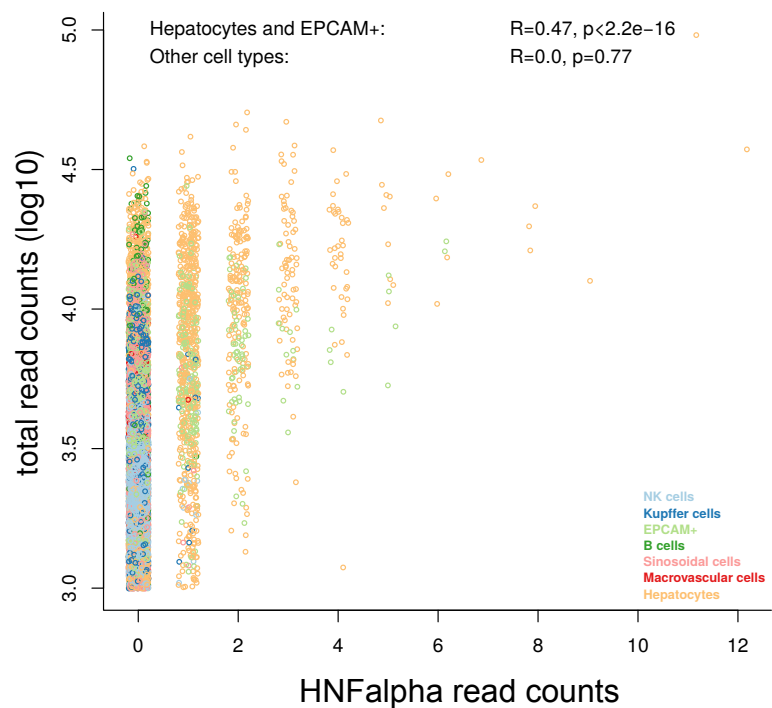

Supplement: Supplementary file 9 — Supplemental Figure 9. [file 41598_2021_88480_MOESM9_ESM.pdf]

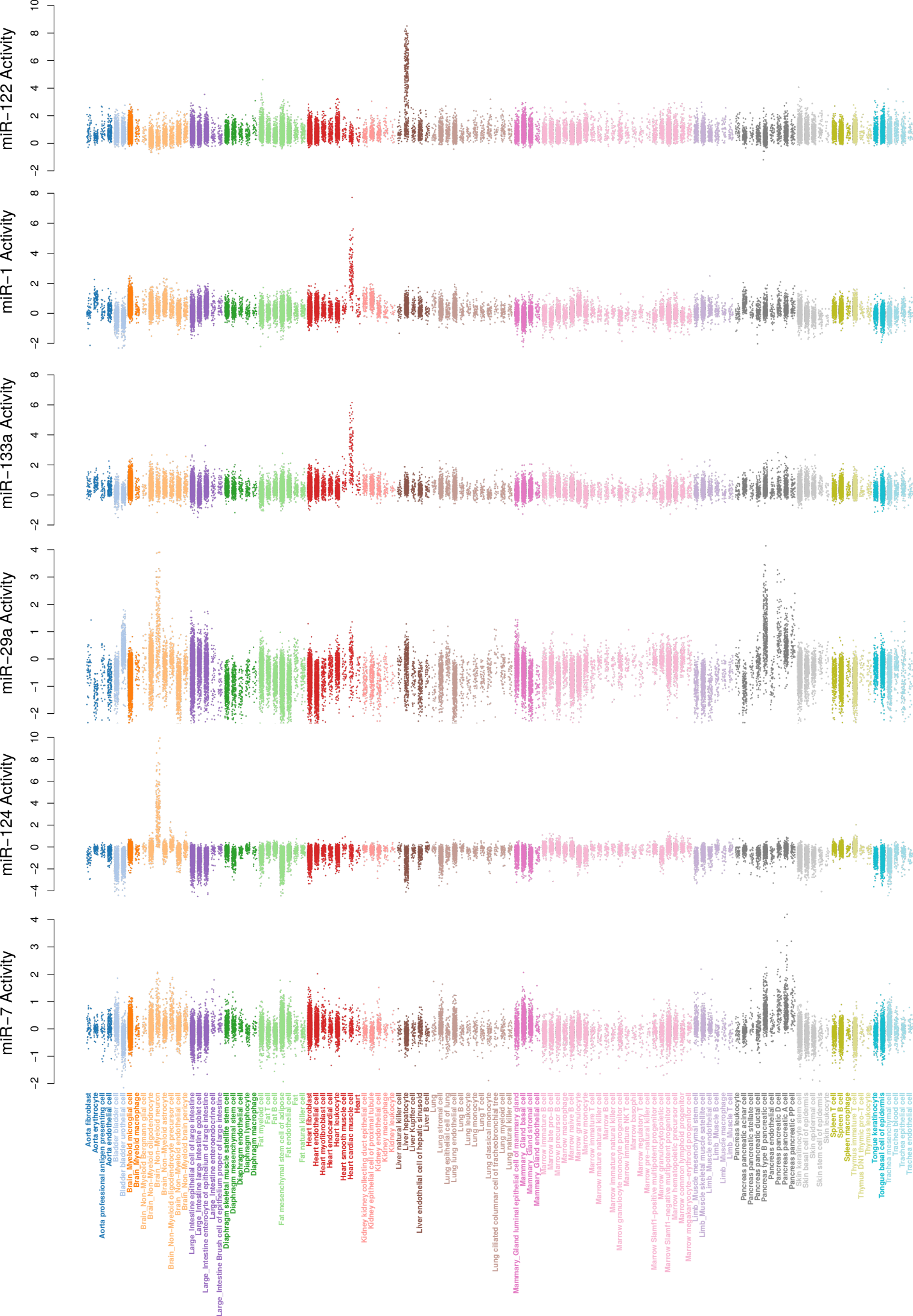

Supplement: Supplementary file 10 — Supplemental Figure 10. [file 41598_2021_88480_MOESM10_ESM.pdf]
