# Supplementary material for: miRNA activity inferred from single cell mRNA expression
Source: Sci Rep. 2021 Apr 28;11:9170. doi: 10.1038/s41598-021-88480-5 (PMC8080788; doi:10.1038/s41598-021-88480-5)
Supplement: Supplementary file 11 — Supplemental Figure 11. [file 41598_2021_88480_MOESM11_ESM.pdf]

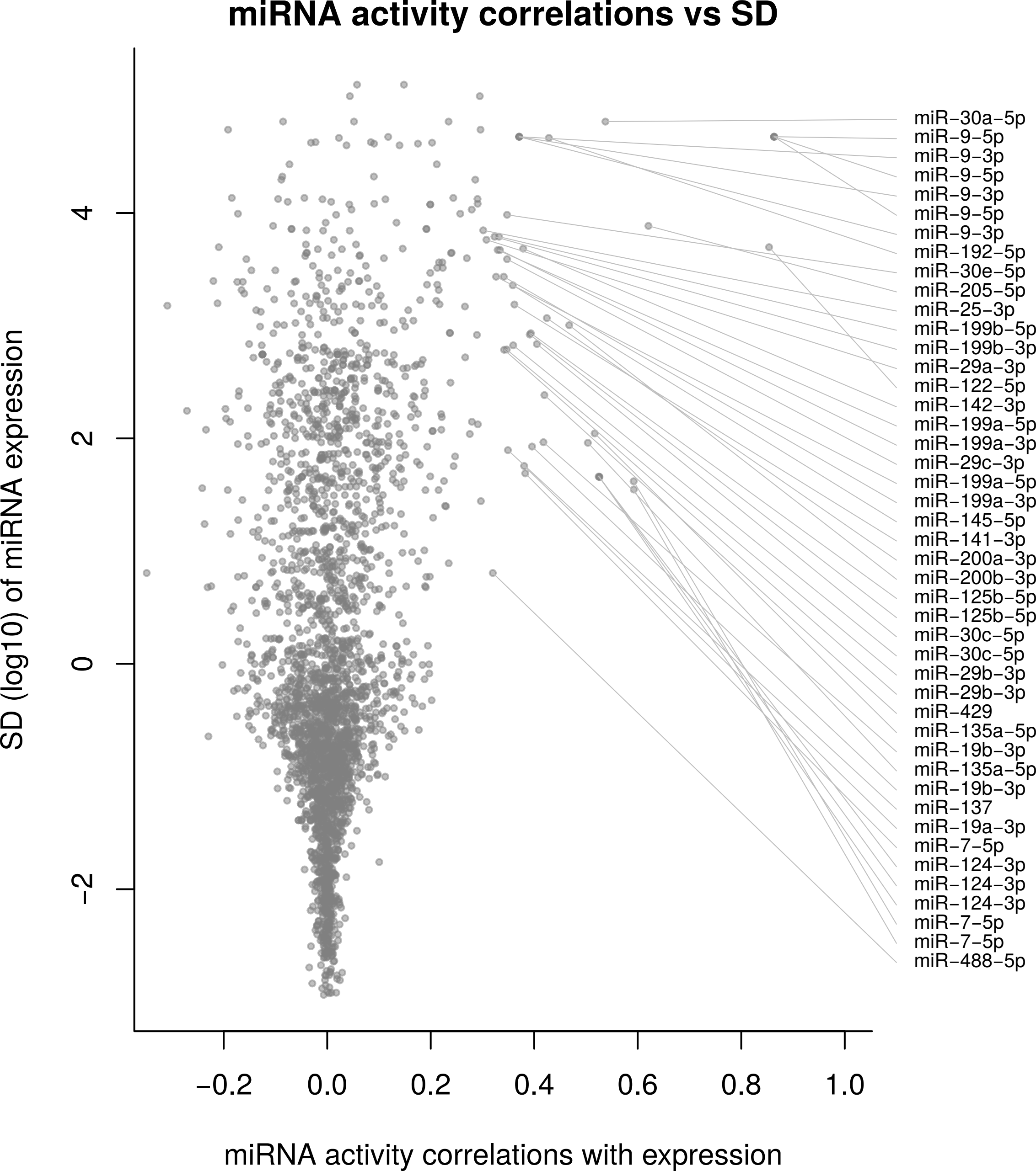

miRNA activity correlations vs SD
SD (log10) of miRNA expression
miRNA activity correlations with expression
-2
0
2
4
-0.2
0.0
0.2
0.4
0.6
0.8
1.0
miR-30a-5p
miR-9-5p
miR-9-3p
miR-9-5p
miR-9-3p
miR-9-5p
miR-9-3p
miR-192-5p
miR-30e-5p
miR-205-5p
miR-25-3p
miR-199b-5p
miR-199b-3p
miR-29a-3p
miR-122-5p
miR-142-3p
miR-199a-5p
miR-199a-3p
miR-29c-3p
miR-199a-5p
miR-199a-3p
miR-145-5p
miR-141-3p
miR-200a-3p
miR-200b-3p
miR-125b-5p
miR-125b-5p
miR-30c-5p
miR-30c-5p
miR-29b-3p
miR-29b-3p
miR-429
miR-135a-5p
miR-19b-3p
miR-135a-5p
miR-19b-3p
miR-137
miR-19a-3p
miR-7-5p
miR-124-3p
miR-124-3p
miR-124-3p
miR-7-5p
miR-7-5p
miR-488-5p
