# Supplementary material for: miRNA activity inferred from single cell mRNA expression
Source: Sci Rep. 2021 Apr 28;11:9170. doi: 10.1038/s41598-021-88480-5 (PMC8080788; doi:10.1038/s41598-021-88480-5)
Supplement: Supplementary file 12 — Supplemental Note. [file 41598_2021_88480_MOESM12_ESM.pdf]

Supplemental Material for 'miRNA activity inferred from single cell mRNA expression'

By Morten Muhlig Nielsen[1] and Jakob Skou Pedersen[1,2*]

[1]Department of Molecular Medicine (MOMA), Aarhus University Hospital, Palle Juul-Jensens Boulevard 99, DK-8200 Aarhus N, Denmark

[2]Bioinformatics Research Centre, C.F. Møllers Allé 8, Aarhus University, DK-8000 Aarhus C, Denmark

Supplemental Methods

Modified Rank Sum Statistic

Let $s_1 s_2, \ldots, s_N$ be a list of sequences ranked according to an experimental setting, and let n_i denote the number of observed motifs in s_i. Under the null model, we assume $n_i \sim po(\lambda_i)$, with $\lambda_i = -\ln(1-p)$, where p is the probability of observing at least one motif in the sequence. This follows from the probability mass function of the the Poisson distribution

$$Pr(X = k) = \frac{\lambda^k}{k!} e^{-\lambda};$$

since $p = 1 - Pr(X = 0) = 1 - e^{-\lambda}$ we have $\lambda_i = -\ln(1-p)$.

If we think of motif occurrences as a Poisson process, where our "time axis" is composed of consecutive intervals of length λ_i ordered according to the experimental rank, motif occurrences are now, under the null hypothesis, uniformly distributed on the interval $[0, \lambda.]$, where $\lambda. = \sum_{i=1}^{N} \lambda_i$.
We now calculate a score r_m, corresponding to the mid point of the interval (sequence) in which a motif was observed.

$$r_m = \frac{\sum_{i=1}^{m-1} \lambda_i + \sum_{i=1}^{m} \lambda_i}{2}$$

We associate the score with motif occurrences in the sequence list. Under the null hypothesis, the probability of observing a motif in a sequence is proportional to the interval length, and thus the expectation is that motif scores are uniformly distributed across the whole interval $[0, \lambda.]$. Under the null model, the score for motif occurrences is thus normally distributed with mean $\lambda./2$ and variance $\lambda.^2/12$.
We calculate the test statistic

$$W = \frac{\sqrt{n.}}{\lambda.} \left(\frac{\sum_{i=1}^{N} n_i r_i}{n.} - \frac{\lambda.}{2} \right) \sim \mathcal{N}\left(0, \frac{1}{12}\right)$$

where $n. = \sum_{i=1}^{N} n_i$. The motif correlation p-value is $p = 2[1 - \Phi(|W|)]$.

Brownian Bridge Method

This method is a re-implementation of the method developed by Jacobsen et al. [1] and recently implemented in cWords [2]. Our implementation differs in the calculation of the sequence dependent motif p-values. The method calculates the max value D of a running sum of mean adjusted log scores of the ranked sequence dependent p-values p_i (SSP_i)
$\mathrm{r}_i = r_{i-1} + ls_i - \bar{ls}$,
where $ls_i = -\ln(p_i + \alpha)$ and α is a score dampening factor of 10^{-5}. $\bar{ls}$ is the mean of the log scores.

The running sum has the form of a bridge (starting and ending in 0), and the maximum value is compared to the theoretical distribution of the absolute maximum M of a Brownian bridge under the null model [3]

$$Pr(M \geq m) = 1 - 2 \sum_{k=1}^{\infty} (-1)^k e^{-2k^2 m^2 / n}$$

where n is the number of sequences in the sequence list.

References

[1] Jacobsen, A., Wen, J., Marks, D. S., and Krogh, A. (2010) Signatures of RNA binding proteins globally coupled to effective microRNA target sites.. *Genome research,* **20**(8), 1010–9.

[2] Rasmussen, S. H., Jacobsen, A., and Krogh, A. (2013) cWords - systematic microRNA regulatory motif discovery from mRNA expression data.. *Silence,* **4**(1), 2.

[3] Billingsley, P. (2008) Convergence of probability measures, John Wiley Sons, Inc., 2 edition.

miReact tutorial

Running miReact on Tabula Muris scRNAseq data set

This tutorial will walk through the steps required to produce miRNA activities from the Tabula Muris FACS sorted data set consisting of mRNA expression measures of ~42,000 cells from 18 tissues.

The end result will consist of a matrix of all 16,384 7-mer motifs evaluated for all ~42,000 cells. A subset of the motifs corresponds to miRNA targets by being complimentary to the miRNA seed sites.

The procedure needs to be run on a cumpute cluster with the sbatch queueing system installed.

For convenience, the miReact repository contains a 1000 cell sampled set of the full data set.

Obtaining the miReact software

miReact is available at the github repository at:

https://github.com/muhligs/miReact

You can either use the Git program to clone the directory, or download the directory from the web.

In the following, we assume that you will place the directory on a compute cluster (required) in the ~/temp directory, so that the directory ~/temp/miReact contains the directories code, data, motif.models and seqs. If miReact is placed differently, please adjust the code in the tutorial accordingly.

The software depends on the R packages 'Regmex', 'expm' and 'parallel', so these should also be installed.

In R do:

```
##############################################
# install expm
install.packages("expm")
# install devtools (if needed) to install Regmex from github:
install.packages("devtools")
library(devtools)
install_github("muhligs/Regmex")
##############################################
```

Calculating motif probabilities in sequences

Prior to making a miReact run, we will need to calculate probabilities for observing motifs in sequences.

This is a somewhat heavy process, and requires multiple cores in an R process. For the mouse sequences, on a laptop/desktop with 16 GB of ram and use of 8 cores, it takes around 10 hours.

If you have more cores available on a cluster node, it is advisable to use it. We have run this on up to 36 cores in less than two hours, but this may require >128 GB of memory.

In addition we need a count matrix of the motifs in the sequences.

In R we do:

```r
################################################
# Calculating motif probabilities in sequences
setwd("~/temp/miReact")
# required packages
require(parallel)
require(Regmex)
require(expm)
source("./code/lambdaProbdist.R") # functions required
seqlist <- readRDS("./seqs/mm.utr3.seqlist.rds") # this is the sequences for mouse 3'UTRs.
load("./motif.models/patterns.7mer.Rdata") # these are motif models

# produce and save the probability matrix
# adjust the number of cores accordingly (mc.cores =)
reslist <- mclapply(patterns,
  function(x)unlist(lapply(seqlist,function(y)pd.mrs2(x,y))),mc.cores = 8)
names(reslist) <- Regmex::all.mers(7)[1:length(reslist)]
pval.mat <- do.call(rbind,reslist)
pval.mat[1:5,1:5] # motifs x sequences
dim(pval.mat) # 16,384 motifs x 52,419 3'UTR sequences
saveRDS(pval.mat, file="./motif.models/mm.seqXmot.utr3_mrs_7mer.rds")

# produce and save the count matrix
# adjust the number of cores accordingly (mc.cores =)
counts <- do.call(rbind,mclapply(Regmex::all.mers(7),
  function(x)Regmex:::n.obs.mot(x,seqlist,overlap=FALSE),mc.cores=8))
rownames(counts) <- Regmex::all.mers(7)
dim(counts) # 16,384 motifs x 52,419 3'UTR sequences
seqs <- readRDS("./seqs/mm.utr3.seqs.rds")
colnames(counts) <- seqs$tid
saveRDS(counts, file="./motif.models/mm.seqXmot.counts.utr3_mrs_7mer.rds")
################################################
```

These files are also available at the Synapse repository at:

https://www.synapse.org/#!Synapse:syn22227236

Download these files ('mm.' for mouse runs and 'hs.' for human runs) and place them in the miReact/motif.models directory.

Obtaining and preparing the Tabula Muris data

The Tabula Muris data set can be obtained from

https://figshare.com/articles/Single-cell_RNA-seq_data_from_Smart-seq2_sequencing_of_FACS_sorted_cells/5715040.

Download the FACS.zip file and unpack into ./data/tm

This should produce 18 .csv files corresponding to 18 different tissues.

In R, prepare the expression data set with the following commands:

```r
################################################
# make raw count expression matrix
setwd("~/temp/miReact/data")
lf <- list.files("tm",full.names = T, pattern = "counts.csv")
```

```r
d <- read.table(as.is=TRUE, sep=",", quote = "", header=TRUE, lf[1],row.names = "X..")
dim(d) # 23,433 x 1,638

for(i in lf[2:length(lf)]){
  d <- cbind(d,
  read.table(as.is=TRUE, sep=",", quote = "", header=TRUE, i,row.names = "X.."))
  print(paste(i,dim(d)[2]))
}
dim(d) # 23,433 x 53,760
# save raw expression matrix
saveRDS(d, file="tm_exp_raw.rds")
###############################################
# make an exp file for miReact...
seqs <- readRDS("~/temp/miReact/seqs/mm.utr3.seqs.rds")
idx <- match(gsub("\"","",rownames(d)),seqs$gsym)
# remove genes without 3'UTRs from expression matrix
d <- d[!is.na(idx),]
# rename genes to ensemble geneIDs
rownames(d) <- seqs$gid[idx[!is.na(idx)]]
dim(d) # 18,005 x 53,760 # 18,005 genes used in the analysis
# define library counts
libs <- apply(d,2,sum)
hist(log10(libs),100) # look at distribution of library counts
saveRDS(libs, file="tm_libs_count.rds")
min(libs) # 14
max(libs) # 12,450,458
median(libs) # 426,493.5
# remove cells with less than 1000 reads or more than 5,000,000 reads
sum(libs<1000) # 3795
sum(libs>5000000) # 27
d <- as.matrix(d)
d <- d[,libs>1000&libs<5000000]
median(libs[libs>1000&libs<5000000]) # 464,902
dim(d) # 18,005 genes x 49,935 cells
# normalize colwize expression to 1 and multiply by median
d <- sweep(d, 2, colSums(d), FUN = '/')*median(colSums(d))
d <- log2(d+1) # log transform
saveRDS(d, file="tm_mireact_exp.rds")
###############################################
```

Running miReact on Tabula Muris data

The miReact run should take place on a compute cluster with the sbatch queuing system installed.

The program will start a process for every 20 samples/cells, and thus this data set will spawn around 2,000 processes.

We have prepared a smaller data set with 1000 cells available on Synapse at:

https://www.synapse.org/#!Synapse:syn22227230

That can be used to test the procedure before performing a full run.

Place this file in the data directory, i.e. ~/temp/miReact/data/mm.exp1000downsample.rds

The mireact function creates a working directory under ~/temp/miReact with format:

o[currentDateAndTime]

Inside this directory, there is an

Rscript-[processID].out

file where the process of the run can be followed.

If the miReact software was placed in a different directory than ~/temp this should be stated in the install.dir parameter (install.dir = "path/to/directory")

in R do:

```
################################################
# Run mireact
source("~/temp/miReact/code/mireact.R")
efile <- "~/temp/miReact/data/tm_mireact_exp.rds"
# If running on the smaller test set, do:
efile <- "~/temp/miReact/data/mm.exp1000downsample.rds"
# This following command starts miReact.

# A mail message can be optionally created
# if the 'mail' program is installed on your system.
# This will send a mail at the start and end of the run.
# Please unset this mail parameter (default mail = NULL) if not available or desired.

# If the miReact software was placed in a different directory than ~/temp
# this should be stated in the install.dir parameter.
# Adjust the name of the outputfile (out.file) accordingly.

mireact(exp=efile, species="mm", motifs=7, seq.type="utr3",
out.file="~/temp/miReact/data/mirnaActivity_tm_1000.rds",out.meonly=T,
mail="myEmail@myMail.server", install.dir = "~/temp/miReact")
################################################
```

Now make some plots of the caluclated miRNA activities. Here shown for the reduced 1000 cell data set. Adjust filenames if needed.

```
################################################
# load motif activity matrix
ma <- readRDS("~/temp/miReact/data/mirnaActivity_tm_1000.rds")
# load cell annotations for sample set
a <- readRDS("~/temp/miReact/data/mm.annotations1000downsample.rds")

## FULL SET ONLY ##########
# load full set annotations
a <- readRDS("~/temp/miReact/data/tm.annotation.rds")
# make ma follow annotation order (and contain cells with annotations only)
colnames(ma) <- sub("[.]$","",sub("X.","",colnames(ma)))
ma <- ma[,a$cell]
## END FULL SET ONLY ######

# source functions
source("~/temp/miReact/code/plot.tools.R")

# plot t-SNE of cells colored by tissue
plot(a$tsne1, a$tsne2, col=a$color, main="", pch=20, cex=1, xlab="", ylab="", axes=F)
text(centroid(a[,c("tsne1","tsne2")], a$cluster2),
```

```r
  labels=a$clusternaming[match(unique(a$cluster2), a$cluster2)],
  cex=1, col=1, lheight=0.1, font=2)
  legend(par("usr")[1]+10, par("usr")[3]+4, unique(a$tissue2), col=unique(a$color),
         bty="n", pch="", ncol = 4, cex=0.7, pt.cex = 0,
         text.col = unique(a$color), xpd=T, text.font = 2)
# now plot the activity overlay for miR-122
plot(a$tsne1[order(ma["ACACTCC",])], a$tsne2[order(ma["ACACTCC",])],
  col=colorme(ma["ACACTCC",], ramp = c("cadetblue1", "white", "darkred"),
  numcol=99, quantcol=F)[order(ma["ACACTCC",])], pch=20, cex=1.5,
  xlab="", ylab="", axes=F, main="miR-122-5p activity")
setKey(colorRampPalette(c("cadetblue1", "white", "darkred"))(99), title="",
  pos=c(-0.05,0.06), label=c("low", "", "high"), height=0.05, keylength = 0.25)

# plot the activities as dots
par(mar=c(9.1,5.1,4.1,2.1))
bardot(ma["ACACTCC",], a$tissue2,col = a$color, main="", ylab="miR-122 Activity",
  ylims=c(-1.1,9), labelpos = -0.03, cex=0.1, labelcex=1,
  labeladj=0.45, ylabcex=1.5, labfont=2)
##############################################
```
